# Trick or Treat: An Intrahepatic Intraductal Papillary Neoplasm of the Bile Duct

**DOI:** 10.7759/cureus.43494

**Published:** 2023-08-14

**Authors:** Mirza Rameez Samar, Zainab Abbasi, Bakhtawar Masood, Nida E Zehra, Adeeba Zaki

**Affiliations:** 1 Oncology, Aga Khan University Hospital, Karachi, PAK; 2 Medicine, Aga Khan University Hospital, Karachi, PAK

**Keywords:** low grade, surgical resection, bile duct, intrahepatic, intraductal papillary neoplasm

## Abstract

An intraductal papillary neoplasm involving the biliary tree is an unusual premalignant condition of epithelial origin, identified by its cystic dilatation of the biliary channels. Being a slow-growing tumor, surgery offers the best curative rate, especially in the setting of a low-grade disease. Here, we present a case of a localized, low-grade, intraductal papillary neoplasm of the bile duct (IPNB), residing in the liver, which was treated with resection of the liver lobe. The adjuvant treatment and prognosis highly depend upon the presence of dysplasia or a co-existent invasive malignancy. To the best of our knowledge, being a rare entity, this is the first case to be reported from Pakistan.

## Introduction

Intraductal papillary neoplasm of the bile duct (IPNB) is a type of bile duct tumor that is identified as a precursor lesion to invasive cholangiocarcinoma. It originates from the epithelial cells lining the bile ducts and is characterized by a predominantly papillary growth pattern in dilated bile ducts [1]. It is a relatively rare condition, but its incidence is increasing worldwide, predominantly in East Asia, Europe, and North America [2]. It accounts for more than 30% of the bile duct tumors diagnosed in the Asian population [3]. There are four subtypes of IPNB, namely, pancreaticobiliary, gastric, oncocytic, and intestinal. Interestingly, the intraductal papillary neoplasm of the pancreaticobiliary type is most frequently seen in North American and European countries, whereas those of the intestinal and gastric types are generally found in Asian countries [4].

There are two distinct subgroups of IPNB, which differ in their location and histology. Type 1 IPNB predominantly involves the intrahepatic ducts, whereas type 2 IPNB is more commonly found in extrahepatic locations. Type 1 IPNB has fewer invasive components, while type 2 IPNB demonstrates more complex papillary structures [5]. There is limited data on the incidence of IPNB in many parts of the world, including Pakistan.

Here, we discuss a case of a 63-year-old gentleman from Pakistan, who was incidentally diagnosed with IPNB after undergoing partial hepatic resection for a space occupying liver lesion.

## Case presentation

A 62-year-old gentleman, a resident of Karachi, Pakistan, a civil engineer by profession, having Eastern Cooperative Oncology Group (ECOG) score of 1, and known to have hypertension and dyslipidemia, presented to the outpatient clinic of a tertiary care hospital in Pakistan with complaints of abdominal distension along with abdominal pain for the past three months with no history of fever, nausea, or vomiting. His vital parameters were within normal limits. He denied any history of allergies or addiction. He also denied any history of familial diseases. His systemic examination revealed a distended abdomen with mild tenderness in the right upper quadrant. He got an ultrasound (US) of the abdomen, which showed a suspicious lesion involving the left liver lobe.

**Figure 1:**
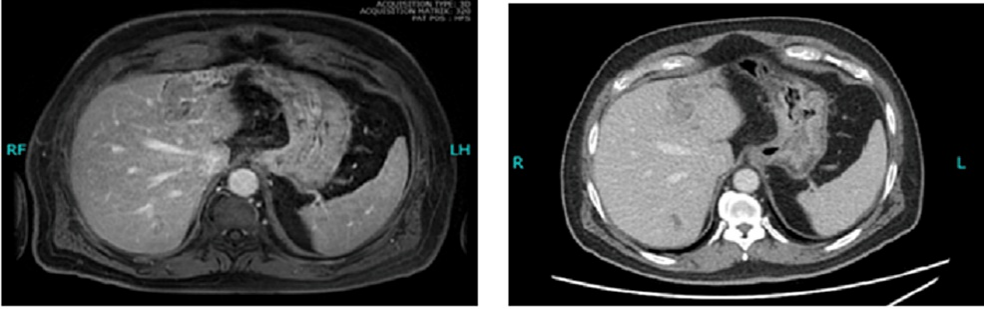
Contrast-enhanced CT showing a heterogeneous infiltrative area involving the left liver lobe in post-contrast (A) and delayed (B) phases.

This was followed by contrast-enhanced computed tomography (CT) of the abdomen, which showed a heterogeneous infiltrative area in the left lobe of the liver with atrophy and intrahepatic biliary dilatation, suggestive of atypical hemangioma versus focal intrahepatic cholangiocarcinoma (Figure 1). His laboratory workup revealed an elevated cancer antigen 19-9 (CA 19-9) level of 144.4 U/ml.

**Figure 2:**
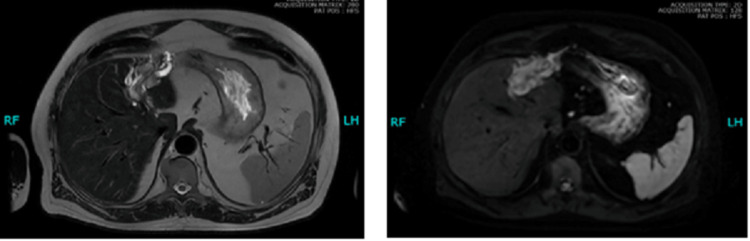
Contrast-enhanced MRI showing a focally enhancing lesion in the left liver lobe appearing isointense in the arterial phase (A) and showing diffuse restriction (B).

Contrast-enhanced magnetic resonant imaging (MRI) of the abdomen demonstrated slight left liver lobe atrophy with a focal enhancing mass lesion of 78 x 32 mm, with multiple tortuous flow voids, arising from the left lobe. A small exophytic component of 15 x 9 mm, arising from this lesion anteriorly, was also appreciated, being isointense in the arterial phase and has a faster washout relative to the rest of the liver with prominent biliary ducts suggestive of local cholangiocarcinoma (Figure 2).

A US-guided liver biopsy was performed, but histopathology showed a scanty tissue sample. After completion of the initial workup, the case was discussed in a multidisciplinary tumor board in our institute, and the patient was advised surgical resection. He subsequently had a left hepatectomy. Intraoperatively, a shrunken left lobe of the liver was appreciated with surrounding parenchymal fibrosis, without any gross ascites and any peritoneal, omental, or bowel deposits suggestive of metastasis. Surgical histopathology showed an intraductal papillary neoplasm of the biliary tree with low-grade dysplasia, confined to the intrahepatic biliary ducts. There was no lympho-vascular invasion or perineural invasion. The bile duct margin and hepatic parenchymal margin were 1 and 0.1 cm, respectively. 

**Figure 3:**
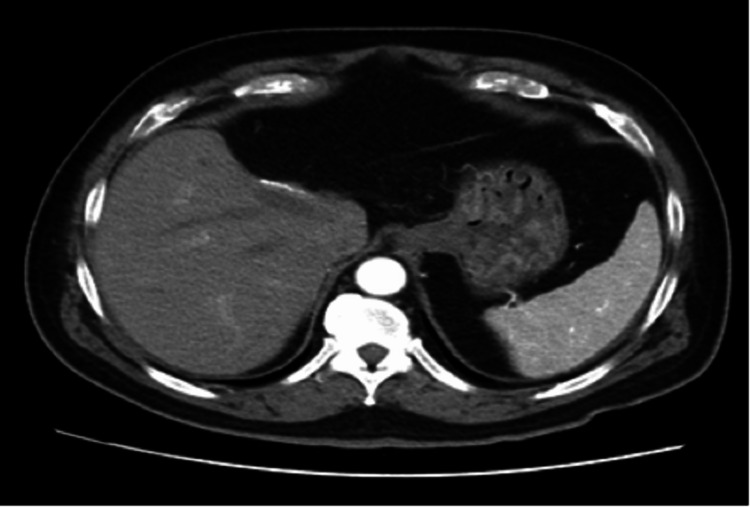
Contrast-enhanced CT showing metallic suturing at the resection site with internal non-visualization of a previously identified left hepatic lobe lesion.

Post-operatively, the patient was discharged in a stable state. After an initial visit for post-operative care on day 7 of his discharge, he was advised to follow up in the clinic with a post-surgical CT abdomen in four weeks, which did not show any residual or recurrent disease (Figure 3). His CA 19-9 levels also declined to 15.2 U/ml. He was then kept on surveillance with repeat CT abdomen advised every six months.

## Discussion

IPNB is a pre-malignant condition that could be referred to as the biliary counterpart of pancreatic intraductal papillary mucinous neoplasm. It was initially called “papillary carcinoma of the bile duct” by Albores-Saavedra, and the term “IPNB” was adopted after being defined by the WHO Classification of Tumors in 2010 [6]. IPNB originates from the epithelial cells lining the biliary channels and could be found anywhere in the biliary tract, i.e., the intrahepatic, peri-hilar, or distal bile duct [7]. It is characterized by a papillary growth pattern or increased secretion of mucin, with resultant cystic dilatation of the biliary tree.

Upfront widespread disease is rare at presentation. In a meta-analysis, compared to the Western population, IPNB in patients residing in Asia was mostly intrahepatic and was less aggressive [7]. Thus, an intrahepatic IPNB can be summarized as a localized neoplasm in the liver with marked biliary duct dilatation having intraluminal filling defects on imaging, papillary-like growth predominantly within the bile ducts on gross examination, and papillary or villous tumors showing fibrovascular cores under microscopy [8].

The clinical spectrum can vary with patients presenting with vague symptoms, such as abdominal discomfort, jaundice, weight loss, and fever. In a review of 28 patients by Wu et al., approximately 46% of the patients were found to have abdominal discomfort as the only symptom at presentation [9]. The diagnosis of IPNB is frequently made through imaging studies, such as magnetic resonance cholangiopancreatography (MRCP) or endoscopic retrograde cholangiopancreatography (ERCP), and histological examination of tissue specimens obtained through biopsy or surgery. MR-based studies are preferred due to their higher sensitivity and the ability of ultrasound or contrast-enhanced CT to overlook small papillary lesions [10]. Histologically, it could be subdivided as IPNB with dysplasia (either low grade or high grade) or invasive carcinoma [11]. Approximately 40-80% of the cases present with an underlying invasive carcinoma [5-12].

Surgical resection is the mainstay treatment for IPNB. Various studies have demonstrated the efficacy of intrahepatic resection, predominantly left hepatectomy with or without biliary duct resection with survival rates ranging from 47% to 68% at five years [8-13]. In a retrospective Korean study involving 146 patients with intrahepatic IPN, of which 17% of cases were of low-grade IPNB, hepatic resection alone resulted in a 10-year survival rate as high as 89% [14]. Although lymph nodal involvement confers to poor prognosis, the therapeutic role of lymphadenectomy remains controversial [13-15].

Despite being a precursor of invasive cholangiocarcinoma, IPNB generally carries a favorable prognosis. Several factors have been associated with early recurrences and worse survival outcomes, including the presence of invasive carcinoma, lymph nodal involvement at diagnosis, R1 resection, lympho-vascular invasion, and extra-ductal spread of tumor [16].

## Conclusions

IPNB is an uncommon, potentially curable biliary tract disease that has propensity to progress to invasive cholangiocarcinoma. The malignant transformation is highly dependent upon the degree of dysplasia. Patients harboring low-grade dysplasia IPNB follow an indolent course and respond well to surgical resection alone with acceptable long-term survival outcomes.
